# Metabolic Detoxification of 2-Oxobutyrate by Remodeling *Escherichia coli* Acetate Bypass

**DOI:** 10.3390/metabo11010030

**Published:** 2021-01-04

**Authors:** Yu Fang, Shuyan Zhang, Jianli Wang, Lianghong Yin, Hailing Zhang, Zhen Wang, Jie Song, Xiaoqing Hu, Xiaoyuan Wang

**Affiliations:** 1 State Key Laboratory of Food Science and Technology, Jiangnan University, Wuxi 214122, China; 7160201025@vip.jiangnan.edu.cn (Y.F.); 6180201052@stu.jiangnan.edu.cn (S.Z.); 8201906111@jiangnan.edu.cn (J.W.); 7180201059@stu.jiangnan.edu.cn (Z.W.); 7190201054@stu.jiangnan.edu.cn (J.S.); xiaoqinghu@jiangnan.edu.cn (X.H.); 2 Zhejiang Provincial Key Laboratory of Resources Protection and Innovation of Traditional Chinese Medicine, Zhejiang A&F University, Hangzhou 311300, China; yinlianghong@zafu.edu.cn; 3 Department of Biological Engineering, College of Life Science, Yantai University, Yantai 264005, China; hlzhang@ytu.edu.cn; 4 Key Laboratory of Industrial Biotechnology, Ministry of Education, Jiangnan University, Wuxi 214122, China; 5 International Joint Laboratory on Food Safety, Jiangnan University, Wuxi 214122, China

**Keywords:** 2-oxobutyrate, acetate bypass, metabolic detoxification, pyruvate oxidase, *Escherichia coli*

## Abstract

2-Oxobutyrate (2-OBA), as a toxic metabolic intermediate, generally arrests the cell growth of most microorganisms and blocks the biosynthesis of target metabolites. In this study, we demonstrated that using the acetate bypass to replace the pyruvate dehydrogenase complex (PDHc) in *Escherichia coli* could recharge the intracellular acetyl-CoA pool to alleviate the metabolic toxicity of 2-OBA. Furthermore, based on the crystal structure of pyruvate oxidase (PoxB), two candidate residues in the substrate-binding pocket of PoxB were predicted by computational simulation. Site-directed saturation mutagenesis was performed to attenuate 2-OBA-binding affinity, and one of the variants, PoxB^F112W^, exhibited a 20-fold activity ratio of pyruvate/2-OBA in substrate selectivity. PoxB^F112W^ was employed to remodel the acetate bypass in *E. coli*, resulting in l-threonine (a precursor of 2-OBA) biosynthesis with minimal inhibition from 2-OBA. After metabolic detoxification of 2-OBA, the supplies of intracellular acetyl-CoA and NADPH (nicotinamide adenine dinucleotide phosphate) used for l-threonine biosynthesis were restored. Therefore, 2-OBA is the substitute for pyruvate to engage in enzymatic reactions and disturbs pyruvate metabolism. Our study makes a straightforward explanation of the 2-OBA toxicity mechanism and gives an effective approach for its metabolic detoxification.

## 1. Introduction

Microbial cells in the processes of growth and production frequently suffer from intracellular toxic metabolites and adverse environmental factors, such as glyoxal [1], methylglyoxal [2], coniferyl aldehyde [3], furfural [4,5] and so on. 2-Oxobutyrate (2-OBA), also known as 2-ketobutyric acid or α-ketobutyrate, belongs to the class of alpha-keto acids known as short-chain keto acids next to pyruvate [6]. As an indispensable intermediate in biological metabolism, 2-OBA is found in all living species ranging from bacteria to humans [7]. However, accumulation of 2-OBA in most microorganisms leads to severe cellular toxicity, including growth arrest or even death [8]. In the biosynthesis process of 2-OBA derivatives, such as l-isoleucine [9,10], l-2-aminobutyric acid [11], 1-propanol [12] and 1-butanol [13,14], the expression level of threonine deaminase catalyzing the breakdown of l-threonine to generate 2-OBA tends to be limited to avoid the toxic accumulation of 2-OBA, which imposes a restriction on the improvement of its downstream flux and target product productivity. This type of biological toxicity not only retards the growth of the fermentation strains, but also blocks the biosynthesis of target products.

Meanwhile, 2-OBA is an important biochemical material used in many fields, such as antiviral agents, food additives and chemical industries [6,7], but its high market price and insufficient supply choke off its widespread application. Nowadays, most 2-OBA products on the market are chemically synthesized from the condensation products of diethyloxalate and ethylpropionate or the condensation products of acetaldehyde [15]. With the development of biotechnology, whole-cell biocatalysis or enzymatic catalysis was applied to produce 2-OBA [16,17]. In addition, *E. coli* strains were genetically engineered to biosynthesize 2-OBA from glucose, and a two-stage fermentation with adoption of a temperature-regulated promoter was designed to avoid cell growth arrest caused by the toxicity of 2-OBA [18]. Although some progress was made on 2-OBA biosynthesis, metabolic toxicity of 2-OBA is still a big problem to limit further improvement of 2-OBA production in *E. coli*. The mechanism of 2-OBA toxicity in microbes has not yet been elucidated systematically, and effective solution for relieving the metabolic toxicity to microbial cells remains to be discovered.

**Figure 1:**
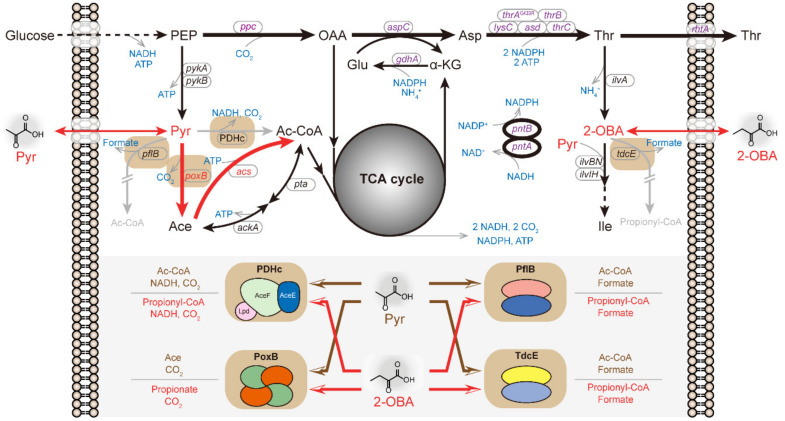
The competition of 2-OBA and Pyr in the l-threonine-overproducing *E. coli* strain TWF115 and the strategy for metabolic detoxification of 2-OBA. In TWF115, the expression levels of the key enzymes relevant to the l-threonine biosynthetic pathway and pyridine nucleotide transhydrogenase (encoded by *pntAB*) are strengthened (bold black lines), and the two genes *pflB* and *tdcE* regarding similar catabolic pathways of pyruvate and 2-OBA are deleted (gray lines). In this study, genes *poxB* and *acs*, forming acetate bypass, were overexpressed in TWF115 under the temperature-controlled promoter pR-pL (bold red lines). The role of PDHc was replaced with the acetate bypass in the PDHc-deficient strain to convert pyruvate to acetyl-CoA. The l-threonine biosynthesis relied on the cofactor supply (NADPH and ATP) mainly from the TCA (tricarboxylic acid) cycle. The four enzymes PDHc, pyruvate oxidase (PoxB) and pyruvate formate-lyase (PflB and TdcE) involved in the catabolism of 2-OBA and pyruvate are displayed with a light brown background. 2-OBA and pyruvate are potentially exchangeable to participate in these enzyme reactions because of their analog structures. PEP, phosphoenolpyruvate; OAA, oxaloacetate; Asp, aspartate; Pyr, pyruvate; Ace, Acetate; AcCoA, acetyl-CoA; α-KG, α-ketoglutarate; Glu, glutamate; Thr, l-threonine; 2-OBA, 2-oxobutyrate; Ile, l-isoleucine.

During the accumulation of 2-OBA, the activity of pyruvate dehydrogenase complex (PDHc) in *E. coli* is shrunken, suggesting a competitive inhibition between 2-OBA and pyruvate [18]. 2-OBA and pyruvate are the common substrates of the three AHAS isozymes (AHAS I (IlvBN), AHAS III (IlvIH) and inactivated AHAS II) in *E. coli* K-12 with different substrate preferences [19,20]. Since 2-OBA and pyruvate are molecular structural analogs, 2-OBA can be considered as a substitute for pyruvate to be involved in the enzymatic reactions which use pyruvate as a substrate (Figure 1). The enzymatic reactions and metabolic pathways in *E. coli* associated with pyruvate were determined [21,22,23]. The key enzymes involved in the principal catabolic branch of pyruvate in the central metabolic pathway include PDHc, pyruvate oxidase (PoxB) and pyruvate formate-lyase (PflB). PflB along with the putative pyruvate formate-lyase (TdcE) may catalyze 2-OBA to generate propionyl-CoA or propionate [24] (Figure 1).

How to enhance intracellular acetyl-CoA pool of the engineered microbes has been a research topic of great concern for a long time [25,26,27,28]. The acetyl-CoA supply in most microorganisms relies mainly on PDHc, with high enzyme activity converting pyruvate to acetyl-CoA [29,30]. The utilization of acetate cooperating with PDHc activity is a resourceful method to increase acetyl-CoA flux [31]. The alternative for catalyzing pyruvate to generate acetyl-CoA is to combine pyruvate oxidase (PoxB) and acetyl-CoA synthetase (ACS), known as acetate bypass, which was used to supplement acetyl-CoA pool for isopropanol production in *E. coli* [32]. PoxB, a thiamin- and flavin-dependent peripheral membrane enzyme in *E. coli,* catalyzes the oxidative decarboxylation of pyruvate to acetate and CO_2_ [23,33]. Similar to pyruvate, 2-OBA can also be the substrate of PoxB and be converted into propionate and CO_2_, but the binding affinity of 2-OBA to PoxB is lower than pyruvate [34] (Figure 1).

In this study, the l-threonine biosynthesis capability of a threonine-overproducing strain of *E. coli* TWF105 was employed to evaluate the biological toxicity of 2-OBA (Figure 1). Considering that the activity of PDHc is inhibited by 2-OBA, the acetate bypass was attempted to be a substitute for the role of PDHc to take charge of the intracellular acetyl-CoA supply. Surprisingly, the acetate bypass alleviated metabolic toxicity of 2-OBA in the absence of PDHc or the *aceE* gene (encoding a subunit of PDHc). Then, single site saturation mutagenesis was executed to enhance pyruvate selectivity of PoxB and attenuate 2-OBA-binding affinity, resulting in PoxB^F112W^. Based on this PoxB variant, the acetate bypass was reconstructed to recharge acetyl-CoA pool and reduce cofactors (NADPH and ATP) used for l-threonine biosynthesis.

## 2. Results and Discussion

### 2.1. Metabolic Toxicity Detection of 2-OBA Using an l-Threonine-Producing E. coli Strain

The sufficient supply of the precursor intermediate oxaloacetate for l-threonine-overproducing strains is vital to l-threonine maximization, whereas the inadequacy of reduced cofactors (NADPH and ATP) and α-ketoglutarate from the TCA cycle is the limitation of l-threonine biosynthesis in engineered *E. coli* strains [35,36]. As shown in Figure 1, the TCA cycle in microbial cells utilizes pyruvate or acetyl-CoA via aerobic catabolism to generate multiple cofactors (NADPH, NADH (nicotinamide adenine dinucleotide) and ATP). The intracellular NADH is converted into NADPH for l-threonine synthesis through pyridine nucleotide transhydrogenase. Additionally, α-ketoglutarate, a TCA cycle intermediate, is the precursor of glutamate biosynthesis which participates in the synthesis of most amino acids in microbes via transamination reactions, including l-threonine biosynthesis. Accordingly, the capability of *E. coli* strains to synthesize l-threonine depends upon metabolic flux into the TCA cycle under the normal conditions of fermentation. Based on the above speculation that metabolic toxicity of 2-OBA results from pyruvate metabolism interference and carbon flux disturbance into the TCA cycle, l-threonine productivity was developed to evaluate metabolic toxicity of 2-OBA in *E. coli*.

Shake flask fermentation of an l-threonine producer *E. coli* TWF105 was performed with the addition of different amounts of 2-OBA to evaluate the inhibitory effect of 2-OBA on l-threonine biosynthesis. Since cell growth arrest in the early stage of fermentation is unfavorable for l-threonine synthesis [35], the time-point of exogenous 2-OBA addition was set at six hours after the beginning of fermentation for the sake of adequate biomass accumulation during the early logarithmic growth phase. To evaluate the inhibitory effect of l-threonine biosynthesis from 2-OBA, the increased l-threonine production due to 2-OBA addition was calculated by subtracting the l-threonine (about 7.4 g/L) secreted before 2-OBA addition; likewise, the inhibition rate of l-threonine biosynthesis was determined on the basis of these increased l-threonine production values. As shown in Table 1, the results demonstrate that l-threonine biosynthesis in TWF105 was inhibited by even 1.0 g/L 2-OBA addition compared with the control (without 2-OBA addition). With the increase of 2-OBA concentrations added, the inhibition rate of l-threonine biosynthesis increased and reached its maximum value (88.79%) with 5 g/L 2-OBA addition. Further increase of the inhibition rate to l-threonine productivity was not observed, even with 10 g/L 2-OBA addition. This could be attributed to two reasons, namely, the secretion of intracellular l-threonine that accumulated, and the water evaporation from the fermentation broth, resulting in about 1.2 g/L of extracellular increased l-threonine. 2-OBA can be obtained by a one-step enzyme reaction of threonine deaminase from l-threonine [37], therefore, TWF105 could be developed for uninterrupted production of 2-OBA from glucose as long as it can acquire 2-OBA tolerance and overproduce l-threonine in the presence of 2-OBA.

### 2.2. Alleviation of the Metabolic Toxicity of 2-OBA in the Engineered E. coli Strains

To investigate how 2-OBA causes toxicity to cell metabolism, some of the major metabolic pathways associated with pyruvate or 2-OBA in the cells must be blocked. Therefore, tdcE was deleted from E. coli TWF105, resulting in TWF115, and aceE, en-coding a subunit of PDHc, was deleted from TWF115, yielding TWF115 ΔaceE. TWF105 [35] is an l-threonine producer lacking PoxB and PflB. A temperature-controlled ex-pression vector pFT26 containing promoters PRL and PR was employed for the induced expression of the acetate bypass consisting of PoxB and ACS in these strains. Four dif-ferent acs genes, acsEc from E. coli and its variants acsEc* which is insensitive to acetyla-tion, acsSe from Salmonella enterica and its variant acsSe* which is resistant to acetylation, were cloned into pET26 together with poxB from E. coli, resulting in plasmids pFT26a1B, pFT26a2B, pFT26a3B and pFT26a4B, respectively. The point mutation L641P is the only difference between ACSEc and ACSEc* as well as between ACSSe and ACSSe*. Previous studies showed that L641P variant made ACS insensitive to acetyla-tion [38] and could improve acetate uptake in E. coli [39]. Accordingly, several recom-binant strains were obtained by transformation of these four plasmids, as well as pFT26 into TWF115 or TWF115 ΔaceE. These recombinant strains were used to assess the capability of l-threonine biosynthesis under the toxic stress of 2-OBA.

**Figure 2:**
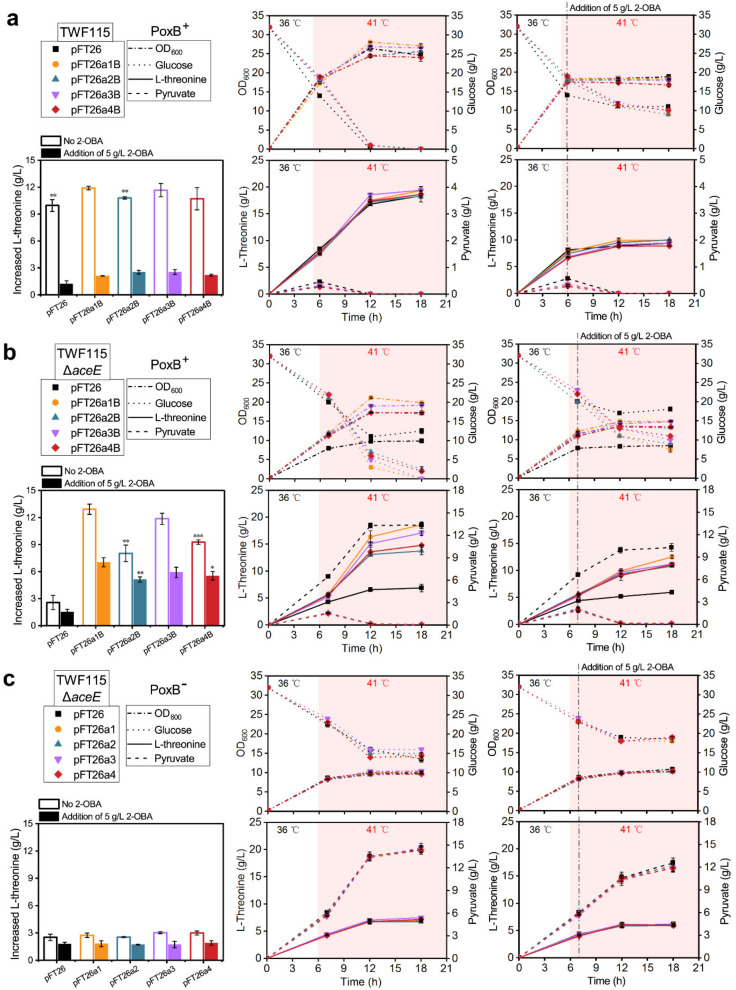
Effects of 2-OBA addition on cell growth (OD_600_), glucose consumption, l-threonine biosynthesis and pyruvate metabolism in different recombinant *E. coli* strains. (**a**) The fermentation of strains TWF115 harboring pFT26, pFT26a1B, pFT26a2B, pFT26a3B and pFT26a4B. The time-point of temperature shift from 36 °C to 41 °C was set at 5 h. (**b**) The fermentation of host strain TWF115 Δ*aceE* harboring pFT26, pFT26a1B, pFT26a2B, pFT26a3B and pFT26a4B. The time-point of temperature shift from 36 °C to 41 °C was set at 6 h. (**c**) The fermentation of host strain TWF115 Δ*aceE* harboring pFT26, pFT26a1, pFT26a2, pFT26a3 and pFT26a4. The time-point of temperature shift from 36 °C to 41 °C was set at 6 h. The time-point for 2-OBA (5 g/L) addition was set at one hour after the temperature shift. The increased l-threonine after the addition of 5 g/L 2-OBA was recalculated by subtracting the l-threonine secreted in the early stage of fermentation. Significance (*p*-value) shown in (**a**,**b**) was evaluated by a two-sided *t*-test, compared to pFT26a1B. The s.d. is shown as black error bars. The two-tailed Student’s *t*-test was used for statistical analysis (*** indicates *p*-value < 0.001, ** indicates *p*-value < 0.01, * indicates *p*-value < 0.05).

To identify the toxic effects of 2-OBA on the l-threonine productivity in these recombinant *E. coli* stains, up-shift temperature from 36 °C to 41 °C was assigned in two fermentation experimental groups (0 or 5 g/L 2-OBA addition). The time-point of 2-OBA addition was set as six or seven hours, one hour after temperature shift, to allow cell growth and biomass accumulation in the early stage of fermentation. The differences between strains were statistically analyzed on the basis of the increased l-threonine after the addition of 5 g/L 2-OBA and the increased value of the control group. After 2-OBA addition, the strains TWF115 harboring pFT26, pFT26a1B, pFT26a2B, pFT26a3B and pFT26a4B abruptly stopped growing, and their glucose consumption significantly slowed down compared with the control group (without 2-OBA addition) (Figure 2a). For the strains TWF115 Δ*aceE* harboring pFT26a1B, pFT26a2B, pFT26a3B and pFT26a4B, the cell growth and glucose consumption also slowed down in the presence of 5 g/L 2-OBA, but they grew better and consumed more glucose than the vector control with or without 2-OBA addition (Figure 2b). For the strains TWF115 Δ*aceE* harboring pFT26, pFT26a1, pFT26a2, pFT26a3 and pFT26a4, similar growth patterns were observed with or without 2-OBA addition, but less glucose was consumed under 2-OBA addition (Figure 2c). In previous studies [40,41], the deletion of PDHc and PoxB in engineered strains was utilized for pyruvate production from glucose; thus, glucose was assimilated and converted into pyruvate in TWF115 Δ*aceE* without PoxB.

As shown in Figure 2a, compared with the control group without 2-OBA addition, l-threonine synthesis in the strains TWF115 harboring pFT26, pFT26a1B, pFT26a2B, pFT26a3B and pFT26a4B was significantly inhibited after the addition of 5 g/L 2-OBA. Surprisingly, the inhibition effect of 2-OBA on l-threonine production in the strains TWF115Δ*aceE* harboring pFT26a1B, pFT26a2B, pFT26a3B and pFT26a4B was obviously weakened, especially pFT26a1B (Figure 2b). The results indicate that ACS_Ec_ and ACS_Se_ are more advantageous to promote l-threonine synthesis compared with their variants ACS_Ec*_ and ACS_Se*_. However, the l-threonine productivity of TWF115 Δ*aceE* was also severely suppressed, even with the intermittent addition of exogenous acetate in the absence of the expression of PoxB (Figure 2c). Moreover, the TWF115 Δ*aceE* harboring pFT26, pFT26a1, pFT26a2, pFT26a3 and pFT26a4 showed indistinctive differences in the increased l-threonine. Without the stress of 2-OBA, the acetate bypass could be a substitute for the role of PDHc in *aceE*-null *E. coli* to restore l-threonine productivity, whereas the individual expression of ACS supplemented with acetate could not produce l-threonine persistently, potentially due to excessive accumulation of pyruvate (above 12.0 g/L) burdening cellular metabolism (Figure 2). Importantly, the acetate bypass not only replaced PDHc to produce acetyl-CoA from pyruvate, but also alleviated the metabolic toxicity of 2-OBA to allow l-threonine biosynthesis in the presence of 2-OBA.

### 2.3. Directed Evolution of PoxB to Improve its Selectivity toward Pyruvate

Similar to PDHc, PoxB is a thiamin diphosphate enzyme that contributes to pyruvate catabolism and aerobic growth in *E. coli* [42]. The activation of the enzyme requires phospholipids or detergents in the presence of the substrate (pyruvate or 2-OBA) and cofactors including thiamin diphosphate, FAD (flavin adenine dinucleotide) and magnesium ions [23]. Although the metabolic toxicity of 2-OBA could be assuaged with the acetate bypass by co-expression of PoxB and ACS, the inhibitory effect of l-threonine synthesis in TWF115Δ*aceE* under 2-OBA addition still existed possibly because of the compatible enzyme activity of PoxB toward 2-OBA and pyruvate (Figure 2b). Therefore, PoxB, with higher selectivity toward pyruvate or without catalytic activity for 2-OBA, is in demand.

**Figure 3:**
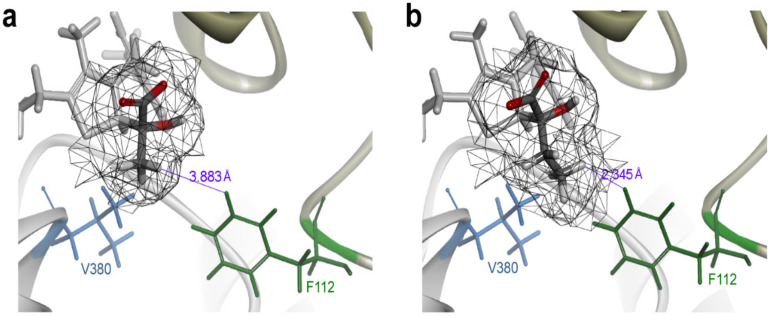
Potential residues for directed evolution of pyruvate oxidase (PoxB) in selectivity for pyruvate. (**a**) Docking simulation of pyruvate covalent conjugate into the substrate-binding pocket. (**b**) Docking simulation of 2-OBA covalent conjugate into the substrate-binding pocket. Residues F112 and V380 at the entrance of the substrate-binding pocket were chosen.

A variant of *E. coli* PoxB, 2-OBA oxidase, was constructed, lacking most catalytic activity for pyruvate by site-directed mutagenesis on the basis of protein sequence alignment [34]. In recent years, molecular docking and virtual screening became reputable methods for efficient recognition of potential mutation sites and directed evolution of enzymes [43]. To seek potential residues of impacting substrate selectivity, the Discovery Studio software was used to dock the pyruvate covalent conjugate or the 2-OBA covalent conjugate into the substrate-binding pocket of PoxB. The analysis showed that residues F112 and V380 at the entrance of the substrate-binding pocket are located adjacent of the alkyl side chains of pyruvate and 2-OBA (within 2–4 Å) and have hydrophobic interactions with the two substrates (Figure 3). The two candidate residues were subjected to the site-directed saturation mutagenesis, followed by mutant screening on triphenyl tetrazolium chloride (TTC) agar plates [44]. As shown in Table 2, the pyruvate/2-OBA activity ratio of wild type (WT) *E. coli* PoxB was 6.23, consistent with previous publication [34]. Compared to the WT PoxB, all variants displayed significantly decreased activities toward pyruvate and 2-OBA, and most variants had lower selectivity for pyruvate. Fortunately, the PoxB^F112W^ variant showed apparently higher activity ratio (20.01) of pyruvate/2-OBA and lost detectable PoxB’s activity for 2-OBA, which implied a substantial enhancement in the pyruvate selectivity. The mutation of the F112 residue better improved PoxB’s selectivity for pyruvate than the V380 residue, in agreement with the results of docking simulation and virtual screening, which showed the distinct distance (3.883 Å and 2.345 Å) between the F112 residue and the alkyl side chains of these two substrates (Figure 3). An L253F mutation involved in the electron transfer to FAD [34] was also introduced into the PoxB^F112W^ variant for the purpose of improving its activity toward pyruvate; however, the resulting PoxB^F112W/L253F^ variant showed a decreased activity ratio of pyruvate/2-OBA in despite of slightly enhanced activity for pyruvate and 2-OBA.

### 2.4. Detoxification of 2-OBA for l-Threonine Biosynthesis by Reconstructing the Acetate Bypass in E. coli

**Figure 4:**
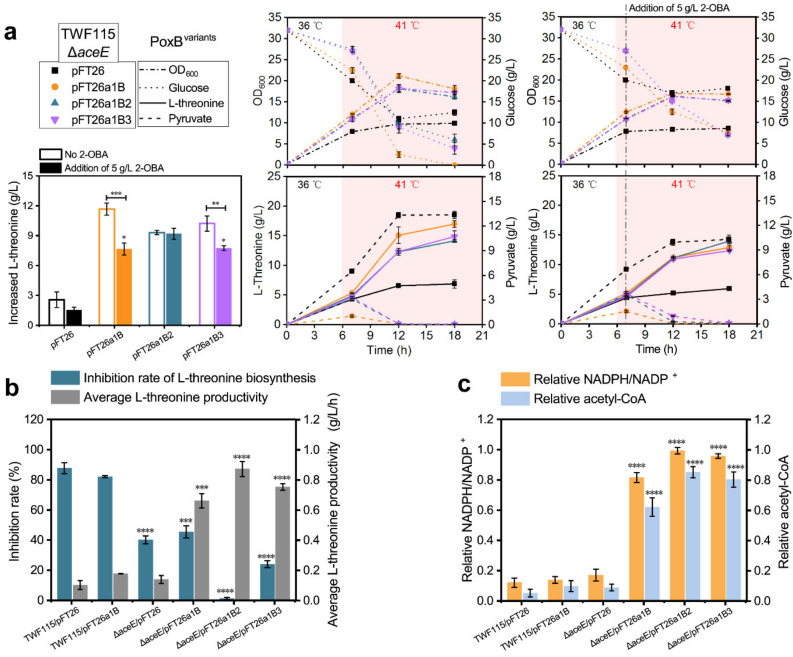
Detoxification of 2-OBA to synthesize l-threonine in engineered strains. (**a**) The fermentation of host strain TWF115 Δ*aceE* harboring the expression vectors of reconstructed acetate bypass with PoxB variants, PoxB^F112W^ and PoxB^F112W/L253F^. The time-point of temperature shift from 36 °C to 41 °C was set as 6 h, and the time point of 2-OBA (5 g/L) addition was one hour after the temperature shift. The increased l-threonine after the addition of 5 g/L 2-OBA was recalculated by subtracting the l-threonine secreted 7 h before. Significance (*p*-value) was evaluated by a two-sided *t*-test, compared to pFT26a1B2. (**b**) Inhibition rate of l-threonine biosynthesis and average l-threonine productivity under the toxic stress of 2-OBA. (**c**) Relative levels of intracellular NADPH/NADP^+^ and acetyl-CoA in the presence of 5 g/L 2-OBA as compared to TWF115/pFT26 with no 2-OBA addition. (**b**,**c**) Significance (*p*-value) was evaluated by a two-sided *t*-test, compared to TWF115/pFT26. The s.d. is shown as black error bars. The two-tailed Student’s *t*-test was used for statistical analysis (**** indicates *p*-value < 0.0001, *** indicates *p*-value < 0.001, ** indicates *p*-value < 0.01, * indicates *p*-value < 0.05).

The two variants PoxB^F112W^ and PoxB^F112W/L253F^ were recruited to reconstruct the acetate bypass by the replacement of WT PoxB, resulting in pFT26a1B2 and pFT26a1B3, respectively. Both plasmids were transformed into TWF115Δ*aceE* as an alternate role of PDHc to produce l-threonine under the toxic stress of 2-OBA. Shake flask fermentation was carried out with the same conditions as the experiments shown in Figure 2b. As shown in Figure 4a, the biomass accumulations of three *aceE*-deficient strains with various types of PoxB increased obviously after adding 5 g/L 2-OBA, as compared with TFW115Δ*aceE*/pFT26. The glucose consumptions of strains with three types of PoxB versus TFW115ΔaceE/pFT26 were ongoing in the presence of 5 g/L 2-OBA, whereas the residual glucose of most strains was not consumed within 18 h, except for TFW115 Δ*aceE*/pFT26a1B2 without 2-OBA addition. As expected, the pFT26a1B2 was able to facilitate TWF115Δ*aceE* to synthesize l-threonine with the highest titer (13.98 g/L) among these vectors when supplemented with 5 g/L 2-OBA (Figure 4a). Relative to the result without 2-OBA addition, the *aceE*-null recombinant strain harboring pFT26a1B2 displayed the best average l-threonine productivity (0.87 g/L/h) and neglectable inhibition rate (1.23%) of l-threonine biosynthesis after 2-OBA addition (Figure 4b). However, the enhancement effect of pFT26a1B2 on l-threonine production of TWF115Δ*aceE* was a little poorer than that of pFT26a1B or pFT26a1B3 in the absence of 2-OBA, due to the decreased catalytic activity of the PoxB^F112W^ variant toward pyruvate. Accordingly, further exploration of PoxB variants with higher activity and higher selectivity for pyruvate is in demand for de novo biosynthesis of 2-OBA and its derivatives.

The metabolic toxicity of 2-OBA in *E. coli* was almost removed completely through the replacement of PDHc with reconstructed acetate bypass based on the PoxB^F112W^ variant. However, the toxicity mechanism of 2-OBA to microbial cells stills needs to be elucidated deeply and requires more data for support. Intracellular NADPH and acetyl-CoA of engineered strains during the process of fermentation with the addition of 5 g/L 2-OBA and no 2-OBA addition were monitored in contrast with the performance of l-threonine biosynthesis by these strains. Relative NADPH/NADP^+^ and relative acetyl-CoA of TWF115/pFT26 and other strains three hours after adding 5 g/L 2-OBA were respectively recalculated by comparing to TWF115/pFT26 without the addition of 2-OBA. In the presence of 5 g/L 2-OBA, the acetate bypass with three types of PoxB in TFW115 Δ*aceE*/pFT26a1B, TFW115 Δ*aceE*/pFT26a1B2 and TFW115 Δ*aceE*/pFT26a1B3 enabled cells to maintain higher intracellular NADPH/NADP^+^ and acetyl-CoA by NADPH and acetyl-CoA quantification (Figure 4c). Our result showed that the changes of relative NADPH/NADP^+^ and relative acetyl-CoA were consistent with that of the l-threonine processivity of each strain affected by the toxicity of 2-OBA (Figure 4b,c). Moreover, the host strain TWF115 Δ*aceE* demonstrated strikingly higher relative NADPH/NADP+ and relative acetyl-CoA and a lower inhibition rate of l-threonine biosynthesis than the host TWF115 after 5 g/L 2-OBA addition. Additionally, the extracellular pyruvate secreted by TWF115 Δ*aceE* during cell growth was reabsorbed and converted into acetyl-CoA through acetate bypass after temperature shift (Figure 4a). Intracellular NADPH used for l-threonine biosynthesis was derived from the oxidative catabolism of acetyl-CoA through the TCA cycle, which was verified by the positive correlation between NADPH/NADP^+^ ratio and acetyl-CoA in Figure 4c. Therefore, the speculation about metabolic toxicity of 2-OBA to microorganisms caused by the deficiency of intracellular acetyl-CoA was further supported by our above work.

## 3. Materials and Methods

### 3.1. Bacterial Strains and Plasmids

All bacterial strains, plasmids and primers used in this study are summarized in Table 3 and Table 4. *E. coli* DH5α was employed as the host for plasmid construction and its genomic DNA was used for the gene cloning template. *E. coli* TWF105, which was previously engineered to overproduce l-threonine [35], was used as the starting strain to test the toxicity of 2-OBA. Plasmid pFT26 harboring a thermosensitive circuit *cI*^ts^-P_RL_ was modified from pFT24 by deleting the TetR repressor and the P_LtetO-1_ promoter. Plasmid pFTS was also derived from pFT24 by retaining the p15A replicon and the triclosan-resistant gene, but removing other genetic elements. Briefly, the DNA fragments to be preserved in the plasmid pFT24 were amplified using the primer pair FWS-mF/FWS-mR, digested with *Dpn*I and chemically transformed into the competent DH5α cells, generating the circular plasmid pFTS with overlap terminus.

### 3.2. Plasmids and DNA Manipulation

Plasmid construction was accomplished with ClonExpress II One Step Cloning Kit (Vazyme, Nanjing, China), together with conventional molecular biology techniques. To construct expression plasmid pFT26-*poxB*, the gene *poxB* was amplified from *E. coli* genomic DNA with the primer pair *poxB*-PR-F/*poxB*-*Eco*RV-R and inserted into MCS2 (Multiple Cloning Site 2) of pFT26. Genes *acs_Ec_* and *acs_Se_* encoding acetyl-CoA synthetase from *E. coli* and *Salmonella enterica*, together with genes acs_Ec_^*^ and acs_Se_^*^ encoding acetyl-CoA synthetase variants resistance to acetylation, were placed respectively under the P_RL_ promoter of pFT26-*poxB*, yielding pFT26a1B, pFT26a2B, pFT26a3B and pFT26a4B. Similarly, those four genes encoding acetyl-CoA synthetase and the *sac*I-digested pFT26 were assembled to generate pFT26a1, pFT26a2, pFT26a3 and pFT26a4, respectively. The gene *poxB* was amplified with primers appended a PJ23101 promoter, and inserted into the plasmid pFTS, resulting in pFTS-*poxB*.

Mutant hosts were prepared using the CRISPR-Cas9 system [45] to manipulate the genome of *E.coli* strains. The l-threonine producer TWF105 was constructed in our previous publication [35] and its *poxB*, *pflB*, *ldhA* and *adhE* genes were removed from TWF001. To close down the main catabolism pathway of pyruvate or 2-OBA in *E. coli*, both *tdcE* and *aceE* genes were deleted from TWF105, resulting in the strain TWF115. In detail, the plasmid pTargetF-*tdcE* targeting the *tdcE* locus with N_20_ sequence was constructed by PCR using template plasmid pTargetF and primers *tdcE*-sg-F/*tdcE*-sg-R. The editing template fragment with two homologous arms corresponding to both ends of the *tdcE* locus was achieved by overlap extension PCR. Then, the template fragment and pTargetF-*tdcE* were merged together and transformed into the strain TWF105 harboring pCas plasmid on electroporation instruments. After incubating at 30 °C for 1 h, the cell culture was concentrated and plated on Luria–Bertani (LB)-agar with the addition of kanamycin (50 mg/L) and spectinomycin (50 mg/L). The *tdcE*-deficient colonies were screened and verified using the primers *tdcE*-up-F/*tdcE*-up-R, and the pTargetF-*tdcE* plasmid was cured by addition of 0.5 mM IPTG. For deletion of *aceE*, the same procedure was carried out using the *tdcE*-deficient TWF105 harboring pCas as the starting strain. Similarly, these four genes (*poxB*, *pflB*, *tdcE* and *aceE*) were also knocked out in *E. coli* MG1655, resulting in MGF01, which served as the host for the directed evolution and mutational screening of PoxB.

### 3.3. Culture and Fermentation Conditions

Luria–Bertani (LB) medium and LB agar plates were used for routine biomolecule manipulation involving gene cloning and recombinant strain construction. The flask fermentation of l-threonine production was carried out in 500 mL baffled shake flasks containing 30 mL fermentation medium (35 g glucose, 15 g (NH_4_)_2_SO_4_, 7.46 g KH_2_PO_4_, 2 g yeast extract, 2 g citrate, 2 g MgSO_4_ 7 H_2_O, 5 mg FeSO_4_ 7 H_2_O, 5 mg MnSO_4_ 4 H_2_O, 0.1 g thiamine-HCl and 20 g CaCO_3_ per liter, pH 7.1), which was modified on the grounds of the published medium formula [35]. For l-threonine biosynthesis trials with or without the addition of 2-OBA in flask, seed strains were streaked on LB agar plates and cultivated for 12 h or 18 h at 36 °C. The seed lawn on plates was scraped into 50 mL STF seed medium (10 g sucrose, 20 g tryptone, 5 g yeast extract, 15 g (NH_4_)_2_SO_4_, and 1 g MgSO_4_ per liter, pH 7.3) [35] and grown for 5 h or 7 h at 36 °C and 200 rpm. Then, the seed culture of 5 mL volume was inoculated into working flask with the initial optical density (OD_600_) 0.2 and fermented at 200 rpm, 36 °C or 41 °C. The commercial 2-OBA purchased from Macklin Technology Co., Ltd. (Shanghai, China) was dissolved into deionized water to 100 g/L and neutralized to pH 7.0 using potassium hydroxide before adding into the fermentation broth. Samples were periodically collected in the process of fermentation. To maintain plasmid stability, appropriate amounts of antibiotics were supplemented with the concentrations of kanamycin, 50 mg/L, spectinomycin, 50 mg/L and triclosan 0.9 mg/L.

### 3.4. Molecular Docking Simulation

The crystal structure of *E. coli* PoxB (PDB ID: 3EY9) was downloaded from the RCSB protein data bank (http://www1.rcsb.org/) and was utilized for the docking simulation of substrate analogs (pyruvate and 2-OBA). The chemical structures of pyruvate and 2-OBA were drawn and converted into their three-dimensional forms using Discovery studio 2016 Client and were sequentially spliced with the molecule of thiamine diphosphate (ThDP) to generate two covalent binding products. These two small molecule products were automatically processed into ligand molecules by the Prepare Ligands program of Discovery studio according to the predefined setting. The preparation of receptor proteins involved the elimination of all bound water molecules, the restoration of hydrogen atoms and the application of a CHARMm force field. The molecular docking between ligand molecules and receptor proteins was conducted using the CDOCKER tool of Discovery studio. The active pocket was composed of amino acid residues around the ThDP (thiamine phosphate) molecule from the original crystal structure of PoxB. The results of the obtained conformations showed that the spatial position of two ligand molecules and hydrogen bond interactions between carbonyl oxygen and the receptor were considered as reasonable.

### 3.5. Library Screening of PoxB Variants

MGF01, which lacks the largely catabolic capability of pyruvate and 2-OBA, was used as the host for PoxB variant screening. Based on pFTS-*poxB* containing a wild-type *poxB* gene with constitutive expression, site-directed saturation mutagenesis of PoxB was carried out. In detail, the plasmid pFTS-*poxB* was used as the PCR template to amplify using whole-plasmid PCR with Phanta Max Super-Fidelity DNA polymerase (Vazyme, Nanjing, China). The primer pair *poxB*-F112-mF/*poxB*-F112-mR containing compatible bases was chemically synthesized by GENEWIZ, Inc. (Suzhou, China) to mutate the F112 residue of PoxB, and the primer pair *poxB*-V380-mF/*poxB*-V380-mR containing compatible bases was chemically synthesized to mutate the residue V380 of PoxB. After gel electrophoresis evaluation and *Dpn*I digestion, the PCR products were directly transformed into the competent DH5α cells and plated on LB agar plates. All colonies grown overnight on the plate were collected into a test tube containing 5 mL LB medium and incubated at 37 °C for 10 h, resulting in PoxB variant library.

For library screening, these plasmids were extracted and introduced into competent MGF01 by electrotransformation, followed by screening on 2,3,5-triphenyl tetrazolium chloride (TTC)-containing LB agar plates. According to the protocol reported previously [44], the MGF01 colonies carrying no-load vector pFTS were recognized as white colonies on TTC-containing LB agar plates supplemented with 20 mM pyruvate or 20 mM 2-OBA, while the MGF01 colonies carrying pFTS-*poxB* were red. These red colonies harboring the PoxB variant library were transferred from TTC-containing plates supplemented with pyruvate to TTC-containing plates supplemented with 2-OBA, and grown overnight at 37 °C. The white or slightly red colonies on the 2-OBA plates were selected as candidates to further assay enzyme activity. In the experiments described above, 0.9 mg/L triclosan was added to maintain plasmid stability.

### 3.6. PoxB Activity Assays

Activity assays of PoxB and its variants were performed on a Cytation 5 microplate reader (Biotek, Winooski, VT, USA) to compare their activities for pyruvate and 2-OBA. The crude enzyme extracts of the host strain MGF01 carrying expression plasmids were prepared according to the protocol published previously [44]. After ultrasonic treatment at 4 °C for 2 min and centrifugation at 13,000× *g* and 4 °C for 20 min, the supernatants including activated enzyme extracts were obtained. The diluted extract (0.4 mL) was added into a reaction mixture (0.5 mL) of PoxB activity assays consisting of 0.2 M sodium phosphate buffer (PH 6.0), 2% Triton X-100, 20 mM MgCl_2_, 0.2 mM sodium thiamine pyrophosphate and either 100 mM pyruvate or 100 mM 2-OBA. The mixture was incubated at room temperate for 10 min to activate these resting PoxB, followed by the addition of 0.08 M sodium ferricynanide (0.1 mL) as the electron acceptor [34]. These reacting mixtures on 96-well plates were used to record the decreased rate of absorbance at 450 nm within 2 min, and the enzyme activities of PoxB and its variants with pyruvate or 2-OBA were calculated as described previously [44]. The total protein concentrations in the cell extracts were quantified with Nanodrop 2000 (Thermo Fisher Scientific, Wilmington, MA, USA) for normalization across samples.

### 3.7. Extracellular Metabolite Analysis

Cell growth was monitored by measuring the optical density at 600 nm (OD_600_) with a UV-1800 spectrophotometer (Shimadzu, Japan). The real-time glucose concentrations in fermentation broths were detected using a biosensor analyzer (SBA-40C, Biology Institute of Shandong Academy of Sciences, Shandong, China). Samples of 1 mL of cell culture were collected and centrifuged at 13,000× *g* for 10 min, and the supernatants of the culture broths were used for extracellular metabolite analysis. The extracellular organic acids, such as 2-OBA and pyruvate, were determined by high-performance liquid chromatography (HPLC) on an Agilent 1200 or 1260 series instrument (Agilent Technologies, Santa Clara, CA, USA) with Aminex HPX-87H column (300 mm × 7.8 mm; Bio-Rad Laboratories, Hercules, CA, USA). The analysis parameters were set as follows: 10 μL injection volume, mobile phase (5 mM sulfuric acid) at a flow rate of 0.6 mL/min, column temperature at 55 °C and DAD detector at emission wavelengths 210 nm. The determination of l-threonine was performed as described previously [35].

### 3.8. Intracellular NADPH and Acetyl-CoA Quantification

Bacterial cells after 8 or 9 h of cultivation in flasks were harvested by centrifugation at 8000× *g* for 10 min at 4 °C. The culture supernatant was thrown away and the cells were washed once with 30 mL ice-cold PBS (phosphate buffered solution) buffer (pH 7.4) solution to remove extracellular metabolites. The cell pellets were quickly frozen and stored in liquid nitrogen until processing. The intracellular levels of NADPH and NADP^+^ were quantified using Coenzyme II NADP(H) Content Assay Kit (Cat# BC1100, Solarbio, Beijing, China). According to the manufacturer’s instructions, NADPH and NADP^+^ were extracted from bacterial cells using basic and acidic extraction reagents, respectively. The oxidized thiazole blue (MTT) was reduced to formazan by NADPH through the hydrogen transfer of phenazine methyl sulfate (PMS). Consequently, the intracellular NADPH could be determined by the absorbance difference at 570 nm. The NADP^+^ level was quantified using glucose-6-phosphate dehydrogenase, which converts NADP^+^ to NADPH.

The measurement of acetyl-CoA level was carried out according to the protocols of Acetyl-CoA Content Assay Kit (Cat# BC0980, Solarbio, Beijing, China). Malate dehydrogenase catalyzes the reversible oxidation of malate to generate oxaloacetate and NADH, while citrate synthase condenses oxaloacetate and acetyl-CoA to form citrate and CoA. The acetyl-CoA level was determined via the formation rate of NADH through the coupled reaction between malate dehydrogenase and citrate synthase. The quantification of NADH was further performed by spectrophotometry on the basis of the ultraviolet absorbance (340 nm).

### 3.9. Statistical Analysis and Reproducibility

All data were indicated as mean ± s.d. The two-tailed Student’s *t*-test and paired sample analysis were used for statistical analysis through SPSS statistics software. Each experiment was repeated at least three times.

## 4. Conclusions

Our study provides an approach for 2-OBA detoxification to engineer *E. coli* production for the first time. The metabolic toxicity of 2-OBA to microbes was evaluated via the determination of increased l-threonine by an l-threonine-producing strain. Then, the PDHc of this strain was replaced with an acetate bypass to generate acetyl-CoA from pyruvate to alleviate the toxicity of 2-OBA and synthesize l-threonine. The PoxB^F112W^ variant with 20-fold activity ratio of pyruvate/2-OBA was screened to reconstruct the acetate bypass, leading to the detoxification of 2-OBA. Our work could pave the way for the industrial-scale production of 2-OBA and its derivatives in microorganisms.

## Figures and Tables

**Table 1:** l-threonine biosynthesis of the l-threonine producer TWF105 with the addition of various amounts of 2-OBA.

Additional 2-OBA (g/L)	0	1.0	2.5	5.0	10.0
Increased l-threonine (g/L)	10.95 ± 0.66	9.32 ± 0.13	6.18 ± 0.18	1.23 ± 0.29	1.24 ± 0.32
Inhibition rate of l-threonine biosynthesis (%)	0	14.86 ± 1.15	43.53 ± 1.67	88.79 ± 2.26	88.71 ± 2.94

**Table 2:** Relative activities of PoxB and its variants with pyruvate or 2-OBA.

Enzyme	Relative Activity (%)		Activity Ratio (Pyr/2-OBA)
	Pyruvate	2-OBA	
**None**	0	0	-
WT	100 ± 2.51	16.06 ± 0.07	6.23
V380C	33.53 ± 2.35	9.37 ± 0.21	3.58
V380I	18.82 ± 0.52	4.24 ± 0.26	4.44
V380T	10.33 ± 0.99	2.80 ± 0.12	3.69
F112A	5.05 ± 0.12	1.66 ± 0.06	3.04
F112L	8.93 ± 0.25	8.57 ± 0.25	1.04
F112T	1.69 ± 0.36	0.51 ± 0.12	3.31
F112W	18.41 ± 0.16	0.92 ± 0.07	20.01
F112W/L253F	26.88 ± 0.36	3.89 ± 0.12	6.91

**Table 3:** Primers used in this study.

Primer Name	Sequence (5′-3′)
*poxB*-PR-F	ATGGTTGCATGAATTCGATCCCTCCGTCAGATGAACTAAAC
*poxB*-*Eco*RV-R	AGCTGGTACCTCGAGTGATTGCCACCCTTTTTACCTTAGC
*acs(Ec)*-F	TTACCTCTTAATTGGAGCTTATGCCACATATTATTAACATCC
*acs(Ec)*-*sac*I-R	CCACTAGTTCTAGAGAGCTCGCCTACAAACCGTTACCGACTC
*acs(Ec)* *-mF	GTAGTCGAGAAGCCGCTTGAAGAGAAG
*acs(Ec)* *-mR	CTTCTCTTCAAGCGGCTTCTCGACTAC
*acs(Se)*-F	TTACCTCTTAATTGGAGCTTATGCCACATATTATTAACATCCTACAAGGAGAACAAAAGCATGAGCCAAACACATAAACACGC
*acs(Se)*-*sac*I-R	CCACTAGTTCTAGAGAGCTCGGCATTTATGGTTATGACGG
*acs(Se)* *-mF	GTGGTGGAGAAACCGCTCGAAGAGAAG
*acs(Se)* *-mR	CTTCTCTTCGAGCGGTTTCTCCAC
*poxB*-PJ-F	TGCTGAAAGGAGTGGAATTTACAGCTAGCTCAGTCCTAGGTATTATGCTAGCAACCCTCCGTCAGATGAACTAAAC
FWS-mF	GCAGGAAGCAATAACTAGCAT
FWS-mR	ATGCTAGTTATTGCTTCCTGCAATAGACCGAGATAGGGTTGAGTG
*poxB*-F112-mF	GCGAAATTGGCAGCGGCTATNNNCAGGAAACCCACC
*poxB*-F112-mR	ATAGCCGCTGCCAATTTCGC
*poxB*-V380-mF	ATGACGCTATTTTCACCTGTGACNNNGGTACGCCAACGGTG
*poxB*-V380-mR	GTCACAGGTGAAAATAGCGTCAT
*poxB*-sg-F	CAAAACACTCGAATCGGCAGGTTTTAGAGCTAGAAATAGC
*poxB*-sg-R	CTGCCGATTCGAGTGTTTTGACTAGTATTATACCTAGGACTGAGC
*poxB*-up-F	TGGGTAGAGCAGGAAGTGAAAGC
*poxB*-up-R	TACAAACCGTTACCGACTCGCACCTGAATGTGATAACGGTAACAAGT
*poxB*-dn-F	TGCGAGTCGGTAACGGTTTGTAGGCGAAAACAAACTGGCTAAGG
*poxB*-dn-R	TATGGGTTGCGGTTGAATACTG
*pflB*-sg-F	GGTGGTATCAAAATGATCGAGTTTTAGAGCTAGAAATAGC
*pflB*-sg-R	TCGATCATTTTGATACCACCACTAGTATTATACCTAGGACTGAGC
*pflB*-up-F	AATGGTCAATGGGGACTAAACG
*pflB*-up-R	GCCTACAAACCGTTACCGACTCGCACCAGGCTGTGGCTAACTTTTCAT
*pflB*-dn-F	TGCGAGTCGGTAACGGTTTGTAGGCTTTCAACTCGCTGACTAAAGAACAG
*pflB*-dn-R	GGTCACCACTTCCTTCATCAAATC
*tdcE*-sg-F	CTGCGTAAAACCCATAACCAGTTTTAGAGCTAGAAATAGC
*tdcE*-sg-R	TGGTTATGGGTTTTACGCAGACTAGTATTATACCTAGGACTGAGC
*tdcE*-up-F	TCTTCGGATTTACGTGTTCTGG
*tdcE*-up-R	GTTGAGGTGTTGACCGCCTTCTCGCCTTCATACGGTGTATAGTT
*tdcE*-dn-F	CGAGAAGGCGGTCAACACCTCAAC
*tdcE*-dn-R	CCCACGGTATGCGAACTG
*aceE*-sg-F	GCACGCAACGAGCAGGATGGGTTTTAGAGCTAGAAATAGC
*aceE*-sg-R	CCATCCTGCTCGTTGCGTGCACTAGTATTATACCTAGGACTGAGC
*aceE*-up-F	TCCAGTATCAGATTGCCGTCAC
*aceE*-up-R	TGACGCAGGTTCACGCTCAACACCTTCTTCAC
*aceE*-dn-F	TGTTGAGCGTGAACCTGCGTCACCACTTCG
*aceE*-dn-R	TTCACCAGGATTTCGGTCACT

*: mutation insensitive to acetylation.

**Table 4:** Strains and plasmids used in this study.

Designation	Genotype or Description	References
*Plasmids*		
pFT24	A thermal switch vector used in *E. coli*, p15A, Tri^R^	[35]
pFT26	*λcI* (ts), P_RL_::MCS1, P_R_::MCS2, p15A, Tri^R^	This study
pFT26-*poxB*	*λcI* (ts), P_RL_::MCS1, P_R_::*poxB*, p15A, Tri^R^	This study
pFT26a1B	*λcI* (ts), P_RL_::*acs_Ec_*, P_R_::*poxB*, p15A, Tri^R^	This study
pFT26a2B	*λcI* (ts), P_RL_::acs_Ec_^*^, P_R_::*poxB*, p15A, Tri^R^	This study
pFT26a3B	*λcI* (ts), P_RL_::*acs_Se_*, P_R_::*poxB*, p15A, Tri^R^	This study
pFT26a4B	*λcI* (ts), P_RL_::acs_Se_^*^, P_R_::*poxB*, p15A, Tri^R^	This study
pFT26a1	*λcI* (ts), P_RL_::*acs_Ec_*, p15A, Tri^R^	This study
pFT26a2	*λcI* (ts), P_RL_::acs_Ec_^*^, p15A, Tri^R^	This study
pFT26a3	*λcI* (ts), P_RL_::*acs_Se_*, p15A, Tri^R^	This study
pFT26a4	*λcI* (ts), P_RL_::acs_Se_^*^, p15A, Tri^R^	This study
pFTS	A small vector modified from pFT26, p15A, Tri^R^	This study
pFTS-*poxB*	PJ23101::*poxB*, p15A, Tri^R^	This study
pFT26a1B2	*λcI* (ts), P_RL_::*acs_Ec_*, P_R_::*poxB*^F112W^, p15A, Tri^R^	This study
pFT26a1B3	*λcI* (ts), P_RL_::*acs_Ec_*, P_R_::*poxB*^F112W, L253F^, p15A, Tri^R^	This study
pCas	Used for gene knockout in *E. coli*, Kan^R^	[45]
pTargetF	sgRNA, pMB1, Spe^R^	[45]
*E. coli* strains		
DH5α	Wild-type	Lab stock
MG1655	Wild-type	Lab stock
MGF01	MG1655 Δ*poxB*Δ*pflB*Δ*tdcE*Δ*aceE*	This study
TWF001	L-Threonine producing *E. coli* strain	[46]
TWF105	TWF001 Δ*poxB*Δ*pflB*Δ*ldhA*Δ*adhE*	[35]
TWF115	TWF105 Δ*tdcE*	This study
TWF115 Δ*aceE*	TWF105 Δ*tdcE*Δ*aceE*	This study

Abbreviations: Tri, triclosan; Kan, kanamycin; Spe, spectinomycin; R, resistance.

## Data Availability

Data sharing not applicable.
